# Supplementary figures and images for: N-Acetyl cysteine exhibits antimicrobial and anti-virulence activity against Salmonella enterica
Source: PLoS One. 2025 Jan 7;20(1):e0313508. doi: 10.1371/journal.pone.0313508 (PMC11706409; doi:10.1371/journal.pone.0313508)

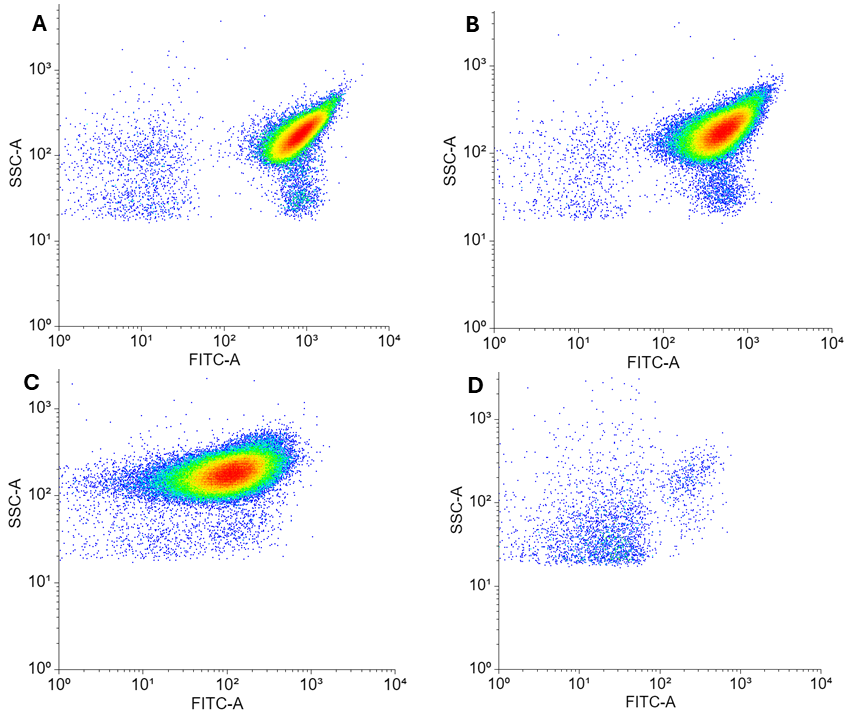

Supplement: S1 Fig — A: control, B: expression at 1.25 mg ml-1 NAC, C: expression at 2.5 mg ml-1NAC and D: expression at 5 mg ml-1NAC. (TIF) [file pone.0313508.s002.tif]

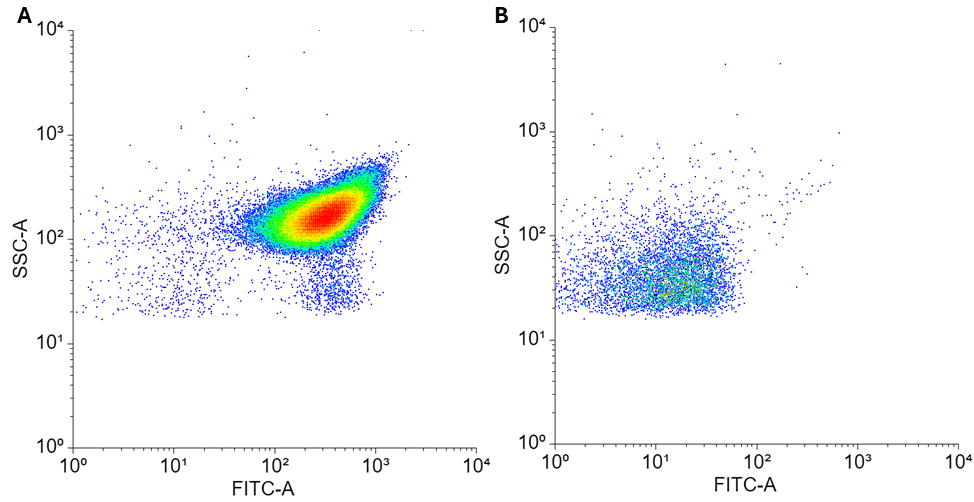

Supplement: S2 Fig — A: Wild type, B: evolved strains. (TIF) [file pone.0313508.s003.tif]
