# Supplementary material for: N-Acetyl cysteine exhibits antimicrobial and anti-virulence activity against Salmonella enterica
Source: PLoS One. 2025 Jan 7;20(1):e0313508. doi: 10.1371/journal.pone.0313508 (PMC11706409; doi:10.1371/journal.pone.0313508)
Supplement: Attachment — Submitted filename: Response to Reviewers .docx [file pone.0313508.s004.docx]

1) **Editor comments:**

When submitting your revision, we need you to address these additional requirements.

**1. Please ensure that your manuscript meets PLOS ONE's style requirements, including those for file naming. The PLOS ONE style templates can be found at**

<https://journals.plos.org/plosone/s/file?id=wjVg/PLOSOne_formatting_sample_main_body.pdf>

and

<https://journals.plos.org/plosone/s/file?id=ba62/PLOSOne_formatting_sample_title_authors_affiliations.pdf>

**Response: Edited**

**2. We suggest you thoroughly copyedit your manuscript for language usage, spelling, and grammar. If you do not know anyone who can help you do this, you may wish to consider employing a professional scientific editing service.**

**Response: A certificate for proof reading is attached.**

**3. The American Journal Experts (AJE) (https://www.aje.com/) is one such service that has extensive experience helping authors meet PLOS guidelines and can provide language editing, translation, manuscript formatting, and figure formatting to ensure your manuscript meets our submission guidelines. Please note that having the manuscript copyedited by AJE or any other editing services does not guarantee selection for peer review or acceptance for publication.**

**Response**: English language was checked by Cairo University Centre for Foreign Languages and Professional Translation. A certificate is attached.

**4. Thank you for stating the following financial disclosure:**

**"This work was partially supported by a visiting fellowship from the Egyptian cultural and educational bureau – Washington, DC."**

Please state what role the funders took in the study.  If the funders had no role, please state: ""The funders had no role in study design, data collection and analysis, decision to publish, or preparation of the manuscript.""

If this statement is not correct you must amend it as needed.

Please include this amended Role of Funder statement in your cover letter; we will change the online submission form on your behalf.

**Response:**

**Part of this work was done during a joint supervision program; the Egyptian cultural bureau paid the monthly stipend for a period of 6 months that’s why we mentioned their partial funding. But the funders had no role in study design, data collection and analysis, decision to publish, or preparation of the manuscript."**

**I mentioned this in my cover letter.**

**5. Please ensure that you refer to Figure 4 in your text as, if accepted, production will need this reference to link the reader to the figure.**

**Response: figure 4 is cited.**

**6. Please include a copy of Table 2 which you refer to in your text on page 5.**

**Response: Table 2 is added to the text.**

**7. Please include captions for your Supporting Information files at the end of your manuscript, and update any in-text citations to match accordingly. Please see our Supporting Information guidelines for more information:** [**http://journals.plos.org/plosone/s/supporting-information**](http://journals.plos.org/plosone/s/supporting-information)**.**

**Response: Supporting information is added to the text.**

8. Please review your reference list to ensure that it is complete and correct. If you have cited papers that have been retracted, please include the rationale for doing so in the manuscript text, or remove these references and replace them with relevant current references. Any changes to the reference list should be mentioned in the rebuttal letter that accompanies your revised manuscript. If you need to cite a retracted article, indicate the article’s retracted status in the References list and also include a citation and full reference for the retraction notice.

**Response: references section is edited.**

**Reviewers' comments:**
Reviewer's Responses to Questions

**Comments to the Author**
1. Is the manuscript technically sound, and do the data support the conclusions?

The manuscript must describe a technically sound piece of scientific research with data that supports the conclusions. Experiments must have been conducted rigorously, with appropriate controls, replication, and sample sizes. The conclusions must be drawn appropriately based on the data presented.

Reviewer #1: Yes

Reviewer #2: Partly

2. Has the statistical analysis been performed appropriately and rigorously?

Reviewer #1: Yes

Reviewer #2: Yes

3. Have the authors made all data underlying the findings in their manuscript fully available?

The [PLOS Data policy](http://c05y1x9s.r.us-east-2.awstrack.me/L0/http:%2F%2Fwww.plosone.org%2Fstatic%2Fpolicies.action%23sharing/1/010f01928a54849a-1e8aacd7-417c-44cd-af8e-789a2d2071f9-000000/HrDgot2kaITeQOZUXtUEk5cx3jc=180) requires authors to make all data underlying the findings described in their manuscript fully available without restriction, with rare exception (please refer to the Data Availability Statement in the manuscript PDF file). The data should be provided as part of the manuscript or its supporting information, or deposited to a public repository. For example, in addition to summary statistics, the data points behind means, medians and variance measures should be available. If there are restrictions on publicly sharing data—e.g. participant privacy or use of data from a third party—those must be specified.

Reviewer #1: Yes

Reviewer #2: Yes

4. Is the manuscript presented in an intelligible fashion and written in standard English?

PLOS ONE does not copyedit accepted manuscripts, so the language in submitted articles must be clear, correct, and unambiguous. Any typographical or grammatical errors should be corrected at revision, so please note any specific errors here.

Reviewer #1: Yes

Reviewer #2: Yes

**5. Review Comments to the Author**

Please use the space provided to explain your answers to the questions above. You may also include additional comments for the author, including concerns about dual publication, research ethics, or publication ethics. (Please upload your review as an attachment if it exceeds 20,000 characters)

**Response to reviewers’ comments**

**Reviewer #1**: This study was designed to investigate the antimicrobial activity of NAC against S. enterica and to verify the potential of NAC as a promising antibiotic for treating S. enterica in combination with other antibiotics. The overall design of the paper is simple and reasonable. **Comments:**

(1) **The basis for selecting Salmonella ATCC 14028 needs to be supplemented.**

**Response: In line 159-163, the discussion section, we explained that we are interested in studying *S. enterica due to the multiple outbreaks and food posisoning associated with Salmonella along with the rapid rate of developing antibiotic resistance .* We conducted similar research on different Gram negative standard stains *(E.coli*, Pseudomonas, Shigella) and clinical isolates but it’s still not published.**

(2) **The method for determining MIC needs to be supplemented, and the reason for not considering MBC as an indicator should be explained.**

**Response: Two folds microdilution technique was used as described in line 210-215.**

**Actually, the concentration mentioned in the manuscript is the MBC as we noticed inhibition of the growth in the well containing (3.125 mg ml-^1^ of NAC) and a visible growth was shown in the well containing (1.56 mg ml-^1^ of NAC). Then we tested concentration between the two values mentioned above (3.1, 3, 2.8. 2.5, 2.3 to 1.5)**

**We found that at concentration of 3 mg ml-^1^of NAC showed a full inhibition of S. *enterica* growth**

(3) **Figure 2 can be further optimized for display, such as using solid or hollow symbols, solid or dashed lines, etc**

**Response: Figure 2 is edited.**

(4) **Is there no color version of Figure 4?**

**Response: unfortunately, no. Plates and media are transparent; bacteria give a white motility zone, that’s why pictures were captured on a dark background for a better contrast to illustrate the motility zone.**

**Reviewer #2**: The present study tries to establish NAC as an adjuvant which can enhance the effect of antibiotics in treatment of Salmonella Enterica infection. The authors derive the minimal inhibitory concentration of various antibiotics in presence and absence of NAC and show that it can be a beneficial addition to the antibiotic pool. They also show that NAC treatment leads to reduced virulence of S. enterica. Though the study demonstrates the positive role of NAC in combatting S. enterica infection, there are major limitations in the study that need to be addressed.

**Major queries:**
**1) The concentration of NAC used in the study is strikingly different from what is reported in one of the previous studies. Ondrej Chlumsky et.al., 2021 (Evaluation of the Antimicrobial Efficacy of N-Acetyl-l-Cysteine, Rhamnolipids, and Usnic Acid—Novel Approaches to Fight Food-Borne Pathogens) showed MIC of NAC to be 12500 μg/mL which is strikingly different from 3 mg/mL used in the present study. Could the authors justify the effect seen with a lower concentration of NAC?**

**Response:**

1) **We think the difference between our MIC values could be due to the starting bacterial load, in our paper NAC was challenged against bacterial concentration of 1x10^5^ CFU ml^-1^ While Chlumsky et.al., 2021 used bacterial concentration of ( OD =)0.08-0.1 that’s approximately 1x10^7^ CFU ml^-1^**
2) **Different strains give different MIC values; we can list multiple papers using NAC against different Gram-negative bacteria. In the above-mentioned paper for Ondrej Chlumsky et.al., 2021 in the second paragraph in the discussion part they said:**

**The efficacy of NAC has been earlier mentioned for the inhibition of biofilm formation, disruption of preformed biofilms (both initial and mature), and reduction in bacterial viability in biofilms of clinical pathogens, such as *Haemophilus influenza, Pseudomonas aeruginosa,* and *Streptococcus pneumoniae* [**[**15**](https://www.mdpi.com/1422-0067/22/21/11307#B15-ijms-22-11307)**,**[**16**](https://www.mdpi.com/1422-0067/22/21/11307#B16-ijms-22-11307)**,**[**19**](https://www.mdpi.com/1422-0067/22/21/11307#B19-ijms-22-11307)**]. Interestingly, these studies demonstrated lower MIC effectiveness against bacterial growth (500–2500 µg/mL) and needed for biofilm reduction (2500–10,000 µg/mL) than MIC evaluated in our study (3130–12,500 µg/mL for bacterial growth and 12,500–100,000 µg/mL for biofilm reduction).**

Moreover we can mention multiple other studies that give vary range of MIC for NAC For Example:

<https://www.ncbi.nlm.nih.gov/pmc/articles/PMC10376233/>

MIC for NAC is 4000 μg/mL

<https://www.ncbi.nlm.nih.gov/pmc/articles/PMC3251652/>

MIC for NAC is 8 mg/ml.

**2) The authors show that administration of NAC leads to decreased expression of virulence genes: hilA and fliC. It is important to understand what this would mean physiologically. Does the reduced virulence lead to less severe symptoms of S. enterica infection? Also, it is recommended that the authors show the expression of other important virulence genes such as invA (assists in the host invasion) and iroB (involved in the formation and transfer of enterobactin).**

**Response:**

**In our previous work we studied the coordinated regulation of virulence in *S. enterica, motility and invasion are tightly coupled*.  fliC is required for the efficient expression and assembly of FliC into the growing flagellar structure*.* Noninvasive Salmonella are not pathogenic, invasion is part of the pathogenesis. The *hilA* gene encodes an OmpR/ToxR family transcriptional regulator that activates the expression of invasion genes, expression of hilA activates the expression of T3-SSS genes including invA. If FliC or HilA expression is affected/ reduced it means the entire infection is aborted.**

**3) The results show that the enhanced strains show a lowered MIC for NAC. How would the authors explain this? And why do the virulence genes in NAC resistant strains respond to NAC treatment?**

**Response:**

**In Figure 2, the evolved strains could tolerate a sub-MIC concentration of NAC, grow to a higher OD compare to the wild type but we were curious to determine what is the change in MIC value for the evolved strains, we found that the MIC values are lower than the wild type may be multiple systems are affected and this needs further investigations like RNA sequencing to check exactly what are the genes involved.**

**May be NAC and other FDA approve substances behave differently than conventional antibiotic, and exposure to sub-MIC is a better option to weaken the pathogen and its virulence factors.**

**Recent papers (**[**https://www.ncbi.nlm.nih.gov/pmc/articles/PMC8721600/**](https://www.ncbi.nlm.nih.gov/pmc/articles/PMC8721600/)**) reported similar findings with treating Pseudomonas with sub MIC concentrations of different antibiotics, MIC post exposure for 10 cycles either remain constsnt, decreased or increases.**

**4) Changes in the WGS profile with NAC treatment would not give a substantial explanation to the changes in virulence. An explanation about the probable mechanism of NAC mediated effects on S. enterica needs to be provided in the discussion section. Also, WGS data need to be validated.**

**Response:**

**WGS showed SNPS in multiple genes some of them are constant in all evolved strains and others are unique per strain. We agree that WGS didn’t give a full explanation but at least gave a picture for the possible genes involved in the interacting pathway of NAC and Salmonella.**

**An extra paragraph is added to the discussion part (line 197 -201)
Minor queries:**

**1) The authors use hilA and fliC promoters driven GFP expression as a readout of virulence. However, GFP expressions are not shown in the manuscript. In addition, flow cytometry plots must be shown in the figure along with the quantification graph.**

**Response: Flow cytometry fplots are added as supplementary documents (S1 Fig, S2 Fig)**

**2) In the abstract, NAC is referred to as an antibiotic and as an adjuvant in another section. It would be better to maintain a uniform terminology. Also, referring to an antioxidant as an antibiotic might need to be reconsidered.**

**Response: edited**

**3) In the discussion section, line 148, it is stated that NAC increases oxidative stress. This misleading statement needs to be corrected.**

**Response: edited**

6. PLOS authors have the option to publish the peer review history of their article ([what does this mean?](https://c05y1x9s.r.us-east-2.awstrack.me/L0/https:%2F%2Fjournals.plos.org%2Fplosone%2Fs%2Feditorial-and-peer-review-process%23loc-peer-review-history/1/010f01928a54849a-1e8aacd7-417c-44cd-af8e-789a2d2071f9-000000/v8EM3Q8ABCMlK8dy4JgKTvHoUKU=180)). If published, this will include your full peer review and any attached files.


If you choose “no”, your identity will remain anonymous but your review may still be made public.


**Do you want your identity to be public for this peer review?** For information about this choice, including consent withdrawal, please see our [Privacy Policy](https://c05y1x9s.r.us-east-2.awstrack.me/L0/https:%2F%2Fwww.plos.org%2Fprivacy-policy/1/010f01928a54849a-1e8aacd7-417c-44cd-af8e-789a2d2071f9-000000/k_DRIuQnLWpmlRf3UV_QCv-f-YI=180).

Reviewer #1: **Yes:** Qingli Dong

Reviewer #2: No
